# Comparison of 27-Gauge to 25-Gauge Vitrectomy in Patients with Tractional Retinal Detachment Associated with Proliferative Diabetic Retinopathy

**DOI:** 10.3390/jcm14072533

**Published:** 2025-04-07

**Authors:** Hiromi Ohara, Tomohiko Torikai, Jun Takeuchi, Tadashi Yokoi, Takashi Koto, Makoto Inoue

**Affiliations:** Kyorin Eye Center, Kyorin University School of Medicine, 6-20-2 Shinkawa, Mitaka, Tokyo 186-8611, Japantomohiko-torikai@ks.kyorin-u.ac.jp (T.T.); jun-takeuchi@ks.kyorin-u.ac.jp (J.T.); tadashi-yokoi@ks.kyorin-u.ac.jp (T.Y.); koto@ks.kyorin-u.ac.jp (T.K.)

**Keywords:** tractional retinal detachment, proliferative diabetic retinopathy, vitrectomy, 27-gauge, 25-gauge, twin duty cycle

## Abstract

**Background/Objectives:** To compare the surgical outcomes of 25-gauge (G) vitrectomy to those of 27G vitrectomy for proliferative diabetic retinopathy (PDR) with a tractional retinal detachment (TRD). **Methods:** Eighty-three consecutive eyes of 71 patients with PDR and TRD that underwent initial vitrectomy at the Kyorin Eye Center from June 2021 to August 2023 and were followed for ≥3 months were studied retrospectively. The surgical outcomes of the 10,000 cut/min (cpm) 25G vitrectomy (25G group, 25 eyes) to that of the 20,000 cpm 27G vitrectomy (27G group, 58 eyes) were compared. **Results:** The preoperative PDR status, surgical procedures, and postoperative outcomes were assessed relative to the surgical success. The 25G group had significantly more eyes with severe PDR (*p* = 0.010), no prior laser photocoagulation *(p =* 0.027), macular detachment (*p* = 0.006), and the use of bimanual technique (*p* = 0.005). However, the operative times and incidence of iatrogenic breaks were not significantly different. The visual acuity improved significantly in both groups at 3 months postoperatively. The primary anatomical success was 88% in the 25G and 97% in the 27G groups (*p* > 0.05). The risk factors for a postoperative retinal detachment were significantly associated with the grade (*p* = 0.042) and type of PDR (*p* = 0.041), the use of perfluorocarbon liquid (*p* = 0.028), and bimanual techniques (*p* = 0.017). **Conclusions:** The high anatomical success for both groups for TRD secondary to PDR indicates that both can be used to treat eyes with PDR. The 27G vitrectomy may reduce the need for bimanual techniques.

## 1. Introduction

Proliferative diabetic retinopathy (PDR) can progress to vitreous hemorrhages, tractional retinal detachment (TRD), combined tractional and rhegmatogenous retinal detachment (RRD), and neovascular glaucoma [1,2]. Pars plana vitrectomy (PPV) is used to treat these complications, and the Diabetic Retinopathy Vitrectomy Study has reported on the benefits of early PPV [1]. The aims of PPV are to remove the vitreous hemorrhage and the retinal traction, create posterior vitreous detachments, remove fibrovascular membranes (FVMs), and treat areas of retinal ischemia [2]. The degree of surgical difficulty varies considerably and depends on the severity and degree of the PDR [2].

The essential step in PPV is to remove the FVMs, which then reduces the traction on the retina. However, releasing the retinal traction during PPV can also induce bleeding and retinal breaks [3]. The procedures and instruments used during PPV have significantly improved, especially the use of microincision vitrectomy systems (MIVS) [4,5,6]. The use of intraoperative intraocular pressure (IOP) stabilization systems, wide-angle endo-illumination, and wide-angle viewing systems has also improved the success rate.

Recently, beveled-tip high-speed, dual-blade, twin-duty cycle vitreous cutters have become available. These cutters have improved the vitrectomy procedures, which, in turn, have expanded the pathologies that can be treated by vitrectomy. There has also been an improvement in the outcomes of vitrectomy to treat severe PDR cases [4,5,6,7,8,9,10,11,12,13,14]. Additionally, the use of the 27-gauge (G) system is associated with fewer cases of postoperative low intraocular pressure (IOP) [7,8] and the need for suturing [9,10,11] compared to the 25-G systems. There are also only minor changes in the corneal topography and astigmatism, which result in rapid postoperative visual recovery [11].

Previous studies comparing the 25G and 27G systems primarily used single-blade cutters. Although the beveled, high-speed, and dual-blade (twin-duty cycle) cutters have been improved, there are only a few reports on using the twin-duty cycle technology for the treatment of PDR [12]. Thus, the purpose of this study was to assess the advantages and risks of using the 27G twin-duty cycle system to treat eyes with PDR. The results were compared to the results of a 25G single-blade cutter system.

## 2. Materials and Methods

This single-center, observational study was approved by the Institutional Review Committee of the Kyorin University School of Medicine (2403-01). It adhered to the tenets of the Declaration of Helsinki. All of the patients received a detailed explanation of the surgical and ophthalmic procedures, and all signed an informed consent form. All of the patients consented to our review of their medical records and their anonymized use in medical publications.

### 2.1. Study Population

**Figure 1:**
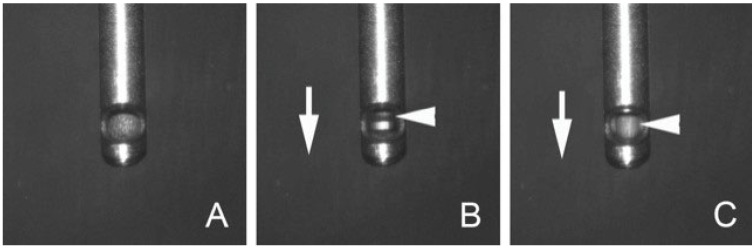
Magnified images of a 27-gauge dual-blade vitreous cutter. (**A**) The opening port of an outer tube is fully opened, and an inner tube of the cutter is located behind the opening port. (**B**) The opening port of the outer tube is closed by the movement of the inner tube forward (arrow). The inner tube has another opening port (arrowhead) that enables fluid or vitreous to be aspirated. (**C**) The opening port of the outer tube is closed by the movement of the inner tube forward (arrow). However, the inner tube has another opening port (arrowhead) that enables fluid or vitreous to be aspirated. This indicates a twin-duty cycle system. On the other hand, a single-blade cutter has no opening port in the inner tube, so when the inner tube is closed, the aspiration is stopped.

This was a retrospective study of patients who had undergone 25G vitrectomy with a single-blade cutter at 10,000 cuts per min (cpm; 25G Group, Alcon Laboratories, Inc., Fort Worth, TX, USA) or 27G vitrectomy with a dual blade cutter at 20,000 cpm (27G Group; Alcon Laboratories, Inc., Fort Worth, TX, USA, Figure 1). All surgeries were performed at the Kyorin University Hospital to treat the TRD, followed by PDR between June 2021 and August 2023. All patients were followed for at least 3 months. Patients were excluded if they had a history of PPV, RRD not followed by PDR, and an exudative or traumatic retinal detachment.

### 2.2. Objectives and Data Collection

The primary outcome was a comparison of the surgical results and the complications of the 25G to the 27G systems. The secondary outcomes were the primary success rates and the identification of factors associated with the success in reattaching the retinal detachment.

The data collected from the medical records were the age at the time of surgery, sex, type of diabetes mellitus, best-corrected visual acuity (BCVA), and IOP (mmHg) at the baseline and at 3 months after the surgery. We also collected information on whether any prior preoperative laser photocoagulation or injections of anti-vascular endothelial growth factor (VEGF) agents had been performed either alone or combined with RRD or neovascular glaucoma, macular detachment, the extent of FVM, and the surgical indications. The extent of the FVM and the surgical indications were determined from the earlier reports [13]. The extent of the FVM was classified as multiple-point adhesions with or without one site plaque-like broad adhesion (Grade 1), broad adhesions in fewer than three sites located from the posterior pole to the equator (Grade 2), broad adhesions in more than three sites located posterior to the equator, or extending beyond the equator within one quadrant (Grade 3), and broad adhesions extending beyond the equator for more than one quadrant (Grade 4) [13].

The surgical indications for PDR included tractional macular thickening, elevation or retinoschisis (Group III), tractional macular detachment (Group IV), and combined traction and RRD (Group V). We injected anti-VEGF agents within 7 days before the PPV in selected eyes [3]. The surgery included the use of 25G or 27G vitrectomy systems, operation time (minutes), use of perfluorocarbon liquid, bimanual technique, incidence of iatrogenic break, and tamponade agent (air, sulfur hexafluoride [SF6], octafluoropropane [C3F8], or silicone oil). We analyzed the optical coherence tomographic (OCT) findings in patients (*n* = 63) whose preoperative OCT images could be used to evaluate the disruption of the external limiting membrane (ELM) or the ellipsoid zone (EZ), and disorganization of the retinal inner layers (DRIL) of the <1 mm area of the macula. Reoperation due to postoperative retinal detachment was also assessed. For patients who received silicone oil tamponade during the initial surgery, primary surgical success was defined as retinal reattachment after successful removal of silicone oil or under silicone oil without recurrent retinal detachment at 3 months. A final surgical failure was defined as the need for additional surgeries to manage recurrent retinal detachment or the presence of residual silicone oil tamponade at the final follow-up. The decimal BCVA was converted to the logarithm of the minimum angle of resolution (logMAR) for the statistical analyses. Poorer visual acuities were graded as counting fingers, 2.0 logMAR units; hand motion, 2.3 logMAR units; light perception, 3.0 logMAR; and no light perception, 4.0 logMAR.

### 2.3. Surgical Techniques

All patients underwent PPV using the 10,000 cpm 25G or the 20,000 cpm 27G Constellation vitrectomy systems (Alcon Laboratories, Inc., Fort Worth, TX, USA). The decision on which vitrectomy system to use was the surgeon’s preference. The duty cycle is the percentage of time the cutter port is open during a cutting cycle. The maximum cutting rate was set at 10,000 cpm in the 25G group or 20,000 cpm in the 27G group. The aspiration pressure was set at 0−650 mmHg. For posterior visualization, the Resight 700 fundus viewing system (Carl Zeiss Meditec AG, Oberkochen, Germany) was used in all cases. Initially, core vitrectomy was performed, and any vitreous hemorrhage was aspirated. The FVM was removed with the vitreous cutter probe, micro-forceps, micro-scissors, or with bimanual techniques. We removed the epiretinal membrane (ERM) or internal limiting membrane that was made visible by injecting brilliant blue G if patients had ERM or macular traction. Panretinal photocoagulation was performed. Phacoemulsification was performed in patients with lens opacities that affected the intraoperative visibility. Air, 20% SF6, 14% C3F8, or silicone oil was used for tamponade when needed.

### 2.4. Statistical Analyses

The characteristics of the retina at the baseline and the surgical details were compared between the two groups according to surgical outcomes using χ^2^ tests, Fisher’s exact tests, or Wilcoxon–Mann–Whitney tests. Dunnett’s tests were used to compare the visual acuity preoperatively and at 1 and 3 months postoperatively. The JMP software, version 18 (SAS Inc., Cary, NC, USA), was used for all statistical analyses, and a *p* < 0.05 was considered statistically significant.

## 3. Results

### 3.1. Preoperative Demographics and Surgical Procedures

The preoperative demographics and surgical procedures of 83 consecutive eyes of 71 patients are shown in Table 1. Twenty-five eyes in the 25G Group and fifty-eight eyes in the 27G Group underwent vitrectomy. The 25G group had a significantly greater number of eyes with severe PDR type (*p* = 0.010), no prior laser treatment (*p* = 0.027), macular detachment (*p* = 0.006), and use of the bimanual technique (*p* = 0.005; Table 1). However, there were no significant differences in age, type of diabetes mellitus, preoperative BCVA, preoperative injection of anti-VEGF agents, preoperative ELM and EZ disruptions, and preoperative DRIL. Preoperative anti-VEGF injection was performed on 14 eyes with no significant difference in the PDR grade (*p* = 0.288) or PDR type (*p* = 0.332). Thirteen of seventy-seven eyes were phakic, and all thirteen eyes underwent lens-sparing vitrectomy; the other sixty-four eyes underwent phaco-vitrectomy.

### 3.2. Comparisons of Postoperative Outcomes Between 25G and 27G Groups

**Figure 2:**
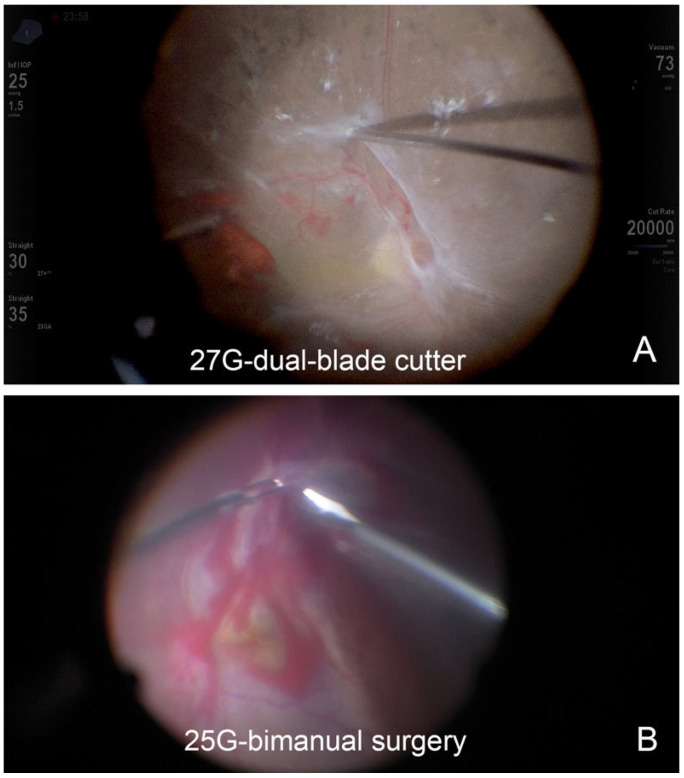
Surgical images during surgery for proliferative diabetic retinopathy with 25-gauge or 27-gauge vitrectomy. (**A**) A fibrovascular membrane is dissected with a 27-gauge dual-blade cutter by inserting the tip of the vitreous cutter beneath the fibrovascular membrane. (**B**) A bimanual technique is used with scissors and forceps of 25-gauge instruments. The fibrovascular membrane is held with the forceps and dissected with scissors in an eye with retinal detachment associated with a tractional membrane complicated by retinal breaks.

We used SF6 in 42 eyes, C3F8 in 12 eyes, and silicone oil in 10 eyes as a tamponade. The operation time and incidence of iatrogenic retinal tears were not significantly different between the two groups, despite the fact that the 25G group had more severe cases of PDR (Table 1 and Figure 2). The operation time in both the 25G and 27G groups was significantly associated with the PDR type (*p* = 0.032, Wilcoxon–Mann–Whitney test), preoperative anti-VEGF injection (*p* = 0.003), use of perfluorocarbon liquid (*p* = 0.016), use of tamponade agent (*p* = 0.001), and complications of iatrogenic breaks (*p* = 0.001). However, there was no significant difference in the other factors.

The median BCVA at the baseline was 1.0 logMAR units with a range of −0.08 to 4.0 logMAR units. The visual acuity was significantly improved to 0.39 logMAR units (range, −0.08 to 2.3 logMAR units; *p* < 0.001) at 3 months after the surgery. This significant improvement was found in both groups (*p* = 0.006 in Group 1; *p* < 0.001 in Group 2).

### 3.3. Primary Success Rates and Risk Factors for Surgical Failure

The primary success rate was 94% for all cases, and it was 88% for the 25G group and 97% for the 27G group (*p* = 0.153). The grade of the PDR (*p* = 0.042), the type of PDR (*p* = 0.041), use of perfluorocarbon liquid (*p* = 0.028), and bimanual technique (*p* = 0.017) were significantly associated with primary failure (Table 2). Otherwise, the age, preoperative BCVA, macular detachment, preoperative anti-VEGF injection, preoperative ELM and EZ disruptions, preoperative DRIL, operation time, iatrogenic break, and tamponade agent were not significantly different between the two groups.

### 3.4. Surgical Failures and Complications

A postoperative retinal detachment was observed in five eyes, and a recurrent retinal detachment caused by the TRD in four eyes (two eyes in the 25G group and two eyes in the 27G group), RRD in one eye (25G group). These five eyes had more severe PDR; four eyes had PDR grade 3, one eye had grade 4, two eyes had type IV PDR, and three eyes had type V. Of the 10 eyes that received silicone oil, 8 eyes had the silicone oil removed, and the retina remained attached. However, a final anatomical success was achieved in 81 eyes (98%), and silicone oil remained in 2 eyes (1 eye in the 25G group and 1 eye in the 27G group).

## 4. Discussion

The 25G high-speed single-blade cutter and the 27G high-speed dual-blade cutter were used for vitrectomy in eyes with a TRD secondary to PDR. The results showed no significant differences in the operative times or incidence of iatrogenic breaks. Despite the higher proportion of severe PDR cases in the 25G group, the primary retinal reattachment rate, operation time, and incidence of iatrogenic breaks were not significantly different between the two groups. Previous studies have similarly reported no significant differences between 25G and 27G vitrectomies in the primary success rate, operative times, and incidence of iatrogenic breaks [14,15,16]. The advancements in MIVS, especially with the bevel-tips and 27G vitrectomy probes, allowed more accurate approaches between the FVM and the retina, which had been difficult with the larger cutters [2]. The smaller port size and the more apical location of the port in the 27G cutters enhanced the removal of FVM and reduced the retinal traction during the removal of FVMs. Furthermore, the 27G cutter can function as a multifunctional tool, e.g., as scissors, picks, and back-flush needles, thus reducing the need to change from the cutter to the other instruments [4]. Previous studies indicated that the 27G system had significantly fewer needs for vitreous forceps [10,14] and diathermy coagulation [10], and more efficient FVM removal [10] than the 25G system. The bimanual technique was used significantly less in the 27G group. Despite these advantages, some studies have reported no significant differences in the operative times [10,14,16], iatrogenic breaks [10,14], need for a tamponade [10,16], or reoperation rates [14,16] between the two groups. The nonsignificant differences in the operation times between vitrectomy with 25G single-blade cutters and 27G dual-blade cutters were likely due to the reduced times for instrument changes and increased vitreous aspiration efficiency with the twin-duty cycle system [17,18]. Furthermore, this has been assumed to be because of the design of the inner tube of the dual-blade cutter. The single-blade vitreous cutter has one port on one side, and an inner tube goes back and forth to open and close the port. On the other hand, the dual-blade cutter had an additional inner opening, which led to a constant aspiration [18]. These differences increased the opening time of the port, and the cutting was from both the forward and backward blades of the cutter [18]. The aspiration rates of the twin-duty cycle cutters for balanced salt solution and vitreous have been reported to be significantly higher than those of single-blade cutters at the maximal cutting rate [18]. On the other hand, the fact that the operative time was comparable between the two groups could be attributed to less severe cases in the 27G group that take less time for FVM removal and more time for vitreous cutting. In the 25G group, the opposite reasons applied. The operation time was significantly associated with PDR type, preoperative anti-VEGF injection, use of perfluorocarbon liquid, use of tamponade agent, and complications of iatrogenic breaks. Further randomized and prospective studies are needed.

Our primary reattachment rate was 94% for TRD secondary to severe PDR. This agrees with earlier MIVS reports of 69.2% to 98.9% [13,16,19]. Severe PDR grade and type were significantly associated with a recurrence of retinal detachment. In contrast, previous studies have identified the poor preoperative BCVA factors as younger age, complication of RRD, and silicon oil tamponade as risk factors for redetachment [13,20,21]. Cases involving severe PDR grades or types and extensive proliferative membranes may require more complex surgical procedures, such as perfluorocarbon or silicon oil tamponade [22,23]. In such cases, a postoperative retinal detachment may develop due to iatrogenic breaks, postoperative rhegmatogenous retinal detachment, or persistent or recurrent traction [24].

There are several limitations in our study. First, this was a single-center, retrospective study, and the selection of the PPV system was dependent on each surgeon’s preference and the preoperative findings. Thus, there may have been a selection bias and variability in each case. Second, the number of patients was small, and meaningful statistical analyses could not be performed, especially in cases of postoperative retinal detachments. Third, the follow-up period was relatively short. Thus, a prospective study with a larger number of cases is needed to validate the findings of this study.

## 5. Conclusions

In conclusion, very good primary and final anatomical success was achieved with both the 25G and 27G vitrectomy for TRD secondary to PDR, with significant visual improvements and no difference in operative time or incidence of iatrogenic break. The use of 27G vitrectomy may reduce the use of bimanual techniques, and the 25G vitrectomy is probably a good procedure for severe PDR.

## Figures and Tables

**Table 1:** Characteristics and surgical details of patients with tractional retinal detachment according to primary surgical outcome.

	Total (*n* = 83)	25G (*n* = 25)	27G (*n* = 58)	*p* Value
Age (years) (median) (range)	50.0 (27–75)	51.0 (27–75)	49.5 (31–69)	0.636 ^§^
Male, *n* (%)	63 (76%)	19 (76%)	44 (76%)	0.989 ^†^
Type 1 diabetes mellitus, *n* (%)	4 (5%)	2 (8%)	2 (3%)	0.921 ^‡^
Preoperative BCVA (logMAR) (median) (range)	1.0 (−0.08–4.0)	1.3 (0.2–4.0)	0.9 (−0.08–2.3)	0.344 ^§^
IOP (mmHg) (median) (range)	15.0 (8–36)	13.0 (9–35)	15.0 (8–36)	0.542 ^§^
PDR grade, *n* (%)				0.129 ^‡^
1	10 (12%)	1 (4%)	9 (16%)	
2	35 (42%)	8 (32%)	27 (27%)	
3	34 (41%)	14 (56%)	20 (35%)	
4	4 (5%)	2 (8%)	2 (4%)	
PDR type, *n* (%)				0.010 *^,‡^
III	39 (47%)	7 (28%)	32 (55%)	
IV	17 (21%)	10 (40%)	7 (12%)	
V	27 (33%)	19 (33%)	8 (32%)	
Preoperative laser photocoagulation, *n* (%)	72 (87%)	19 (76%)	53 (91%)	0.027 *^,†^
Neovascular glaucoma, *n* (%)	3 (4%)	2 (8%)	1 (2%)	0.183 ^†^
Macular detachment, *n* (%)	31 (37%)	15 (60%)	16 (28%)	0.006 *^,†^
Preoperative anti-VEGF injection, *n* (%)	14 (17%)	4 (16%)	10 (17%)	0.889 ^†^
Preoperative ELM disruption, *n* (%)	38 (60%)	16 (73%)	22 (54%)	0.135 ^†^
Preoperative EZ disruption, *n* (%)	47 (75%)	18 (82%)	29 (71%)	0.900 ^†^
Preoperative DRIL, *n* (%)	49 (78%)	20 (91%)	29 (71%)	0.989 ^†^
Operation time (minutes) (median) (range)	117.0 (38–284)	111.0 (68–188)	117.5 (38–284)	0.976 ^§^
Iatrogenic break, *n* (%)	53 (64%)	16 (64%)	37 (64%)	0.986 ^†^
Perfluorocarbon liquid, *n* (%)	28 (34%)	9 (36%)	19 (33%)	0.775 ^†^
Bimanual technique, *n* (%)	25 (30%)	13 (52%)	12 (21%)	0.005 *^,†^
Tamponade agent, *n* (%)	64 (77%)	21 (84%)	43 (74%)	0.315 ^†^

*: *p* < 0.05, ^†^: χ^2^ test, ^‡^: Fisher’s exact test, ^§^: Wilcoxon–Mann–Whitney test, BCVA, best-corrected visual acuity; logMAR, logarithm of the minimum angle of resolution; IOP, intraocular pressure; PDR, proliferative diabetic retinopathy; VEGF, vascular endothelial growth factor; ELM, external limiting membrane; EZ, ellipsoid zone; DRIL, disorganization of retinal inner layers.

**Table 2:** Characteristics and surgical details of patients with re-detachment.

	Re-Detachment (*n* = 5)	Primary Attachment (*n* = 78)	*p* Value
Age (years) (median) (range)	46.0 (41–74)	50.5 (27–75)	0.792 ^§^
Male, *n* (%)	4 (80%)	59 (75%)	0.822 ^†^
Type 1 diabetes mellitus, *n* (%)	1 (25%)	4 (5%)	0.224 ^‡^
Preoperative BCVA (logMAR) (median) (range)	1.2 (0.5–1.7)	0.9 (−0.08–4.0)	0.826 ^§^
IOP mmHg (median) (range)	10.0 (10–16)	15.0 (8–36)	0.085 ^§^
PDR grade, *n* (%)			0.042 *^,‡^
1	0 (0%)	10 (13%)	
2	0 (0%)	35 (45%)	
3	4 (80%)	30 (39%)	
4	1 (20%)	3 (4%)	
PDR type, *n* (%)			0.041 *^,‡^
III	0 (0%)	39 (50%)	
IV	2 (40%)	15 (19%)	
V	3 (60%)	24 (31%)	
Preoperative laser photocoagulation, *n* (%)	3 (60%)	68 (87%)	0.146 ^†^
Neovascular glaucoma, *n* (%)	0 (0%)	3 (4%)	0.538 ^†^
Macular detachment, *n* (%)	5 (46%)	26 (31%)	0.577 ^†^
Preoperative anti-VEGF injection, *n* (%)	2 (40%)	12 (15%)	0.203 ^†^
Preoperative ELM disruption, *n* (%)	5 (100%)	33 (57%)	0.071 ^‡^
Preoperative EZ disruption, *n* (%)	5 (100%)	42 (72%)	0.218 ^‡^
Preoperative DRIL, *n* (%)	5 (100%)	44 (76%)	0.271 ^‡^
Operation time (minutes) (median) (range)	146.0 (96–188)	116.5 (38–284)	0.340 ^§^
25G, *n* (%)	3 (12%)	22 (88%)	0.153 ^†^
Iatrogenic break, *n* (%)	1 (20%)	29 (37%)	0.418 ^†^
Perfluorocarbon liquid, *n* (%)	4 (80%)	24 (31%)	0.028 *^,†^
Bimanual technique, *n* (%)	4 (80%)	21 (27%)	0.017 *^,†^
Tamponade agent, *n* (%)	5 (100%)	59 (76%)	0.101 ^†^

*: *p* < 0.05, ^†^: χ^2^ test, ^‡^: Fisher’s exact test, ^§^: Wilcoxon–Mann–Whitney test, BCVA, best-corrected visual acuity; logMAR, logarithm of the minimum angle of resolution; IOP, intraocular pressure; PDR, proliferative diabetic retinopathy; VEGF, vascular endothelial growth factor, ELM, external limiting membrane; EZ, ellipsoid zone; DRIL, disorganization of retinal inner layers.

## Data Availability

The data presented in this study are available on request from the corresponding author (MI).
